# Video vs Direct Laryngoscopy for Tracheal Intubation After Cardiac Arrest: A Secondary Analysis of the Direct vs Video Laryngoscope Trial

**DOI:** 10.1016/j.chest.2024.12.031

**Published:** 2025-01-11

**Authors:** Amelia L. Muhs, Kevin P. Seitz, Edward T. Qian, Brant Imhoff, Li Wang, Matthew E. Prekker, Brian E. Driver, Stacy A. Trent, Daniel Resnick-Ault, Steven G. Schauer, Adit A. Ginde, Derek W. Russell, Sheetal Gandotra, David B. Page, John P. Gaillard, Lane M. Smith, Andrew J. Latimer, Steven H. Mitchell, Nicholas J. Johnson, Shekhar A. Ghamande, Heath D. White, Kevin W. Gibbs, Jessica A. Palakshappa, Derek J. Vonderhaar, David R. Janz, Micah R. Whitson, Christopher R. Barnes, Alon Dagan, Ari Moskowitz, Vijay Krishnamoorthy, James T. Herbert, Michael D. April, Aaron M. Joffe, Jeremy P. Walco, Christopher G. Hughes, Kipp Shipley, Amelia W. Maiga, Bradley D. Lloyd, Stephanie C. DeMasi, Wesley H. Self, Todd W. Rice, Matthew W. Semler, Jonathan D. Casey

**Affiliations:** a Division of Pulmonary and Critical Care Medicine, Vanderbilt University Medical Center, Nashville, TN; b Department of Biostatistics, Vanderbilt University Medical Center, Nashville, TN; c Department of Anesthesiology, Vanderbilt University Medical Center, Nashville, TN; d Department of Surgery, Vanderbilt University Medical Center, Nashville, TN; e Department of Emergency Medicine, Vanderbilt University Medical Center, Nashville, TN; f Division of Pulmonary, Allergy, and Critical Care Medicine, Department of Medicine, Hennepin County Medical Center, Minneapolis, MN; g Department of Emergency Medicine, Hennepin County Medical Center, Minneapolis, MN; h Department of Emergency Medicine, University of Colorado School of Medicine, Aurora, CO; i Division of Pulmonary, Allergy, and Critical Care Medicine, University of Alabama at Birmingham, Birmingham, AL; j Department of Emergency Medicine, Wake Forest University School of Medicine, Winston-Salem; k Division of Pulmonary, Critical Care, Allergy, and Immunology, Wake Forest University School of Medicine, Winston-Salem; l Department of Anesthesiology, Duke University School of Medicine, Durham, NC; m Department of Emergency Medicine, University of Washington Harborview Medical Center, University of Washington, Seattle, WA; n Department of Anesthesiology and Critical Care Medicine, University of Washington Harborview Medical Center, University of Washington, Seattle, WA; o Division of Pulmonary, Critical Care, and Sleep Medicine, University of Washington, Seattle, WA; p Division of Pulmonary and Critical Care Medicine, Baylor Scott & White Healthcare, Temple, TX; q Division of Pulmonary and Critical Care Medicine, Ochsner Medical Center, LSU School of Medicine New Orleans, New Orleans, LA; r Division of Pulmonary and Critical Care Medicine, LSU School of Medicine New Orleans, New Orleans, LA; s Department of Emergency Medicine, University of Alabama at Birmingham, Birmingham, AL; t Department of Emergency Medicine, Beth Israel Deaconess Medical Center, Boston, MA; u Division of Critical Care Medicine, Montefiore Medical Center, The Bronx, NY; v Uniformed Services University of the Health Sciences, Bethesda, MD; w 14th Field Hospital, Fort Stewart, GA; x Department of Anesthesiology, Creighton University School of Medicine, Omaha, NE

**Keywords:** cardiac arrest, direct laryngoscopy, intubation, video laryngoscopy

## Abstract

**Background:**

Airway management is a critical component of the care of patients experiencing cardiac arrest, but data from randomized trials on the use of video vs direct laryngoscopy for intubation in the setting of cardiac arrest are limited. Current American Heart Association guidelines recommend placement of an endotracheal tube either during CPR or shortly after return of spontaneous circulation, but do not provide guidance around intubation methods, including the choice of laryngoscope.

**Research Question:**

Does use of video laryngoscopy improve the incidence of successful intubation on the first attempt, compared with use of direct laryngoscopy, among adults undergoing tracheal intubation after experiencing cardiac arrest?

**Study Design and Methods:**

This secondary analysis of the Direct vs Video Laryngoscope (DEVICE) trial compared video laryngoscopy vs direct laryngoscopy in the subgroup of patients who were intubated after cardiac arrest. The primary outcome was the incidence of successful intubation on the first attempt. Additional outcomes included the duration of laryngoscopy.

**Results:**

Among the 1,417 patients in the DEVICE trial, 113 patients (7.9%) experienced cardiac arrest before intubation, of whom 48 patients were randomized to the video laryngoscopy group and 65 patients were randomized to the direct laryngoscopy group. Successful intubation on the first attempt occurred in 40 of 48 patients (83.3%) in the video laryngoscopy group and in 42 of 65 patients (64.6%) in the direct laryngoscopy group (absolute risk difference, 18.7 percentage points; 95% CI, 1.2-36.2 percentage points; *P* = .03). The mean duration of laryngoscopy was 48.0 seconds (SD, 37.3 seconds) in the video laryngoscope group and 98.0 seconds (SD, 122.4 seconds) in the direct laryngoscopy group (mean difference, –50.0 seconds; 95% CI, –86.8 to –13.3 seconds; *P* = .004).

**Interpretation:**

Among adults undergoing tracheal intubation after experiencing cardiac arrest, use of video laryngoscopy was associated with increased incidence of successful intubation on the first attempt and shortened duration of laryngoscopy, compared with use of direct laryngoscopy.

FOR EDITORIAL COMMENT, SEE PAGE 1259

**Take-Home Points**

**Study Question:** Does use of video laryngoscopy improve incidence of successful intubation on the first attempt compared with use of direct laryngoscopy among adults undergoing tracheal intubation after experiencing cardiac arrest?

**Results:** Among adults undergoing tracheal intubation after experiencing cardiac arrest, use of video laryngoscopy increased the incidence of successful intubation on the first attempt by 18.7% and shortened the mean duration of laryngoscopy by 50.0 seconds compared with use of direct laryngoscopy.

**Interpretation:** In this study, use of video laryngoscopy for intubation during cardiac arrest improved first-pass success and decreased duration of laryngoscopy when compared with direct laryngoscopy.

Most patients who experience in-hospital cardiac arrest undergo tracheal intubation either during or shortly after CPR.^1^^,^^2^ Tracheal intubation in this setting has been associated with increased odds of difficult intubation resulting from limited time to prepare, suboptimal patient positioning, ongoing CPR, and increased incidence of aspiration.^3^ Failure to intubate on the first attempt has been associated with increased complications such as hypoxemia, aspiration, and dental injury.^4^^,^^5^ Failure to intubate on the first attempt may be particularly harmful for patients undergoing tracheal intubation in the setting of cardiac arrest, because repeated attempts may increase the frequency and duration of interruptions in chest compressions and may delay correction of hypoxemia and hypercapnia, which may be required for return of spontaneous circulation.^6^, ^7^, ^8^ Research suggests that use of video laryngoscopy, compared with use of direct laryngoscopy, increases the incidence of successful intubation on the first attempt among patients undergoing tracheal intubation in the emergency department (ED) or ICU, as well as in the operating room.^9^, ^10^, ^11^

Current American Heart Association guidelines recommend: (1) consideration of an advanced airway, either an endotracheal tube or supraglottic airway, during CPR and (2) early placement of an endotracheal tube after return of spontaneous circulation.^12^ Prior data comparing the effectiveness of video vs direct laryngoscopy in cardiac arrest comes primarily from observational studies and small randomized trials and show mixed results. Two observational studies reported better glottic visualization and a higher incidence of successful intubation on the first attempt with video laryngoscopy compared with direct laryngoscopy.^13^^,^^14^ In contrast, 2 other studies reported no difference in successful intubation on the first attempt.^3^^,^^15^ Two prior single-center randomized trials have attempted to compare video vs direct laryngoscopy during out-of-hospital and in-hospital cardiac arrest and have failed to demonstrate a benefit from video laryngoscopy, but they both included only a small number of highly experienced operators with limited experience with video laryngoscopy.^16^^,^^17^ To evaluate the effect of video vs direct laryngoscopy on outcomes in adults undergoing tracheal intubation after experiencing cardiac arrest, we performed a secondary analysis of the Direct versus Video Laryngoscope (DEVICE) trial, in which patients undergoing emergency tracheal intubation in the ED or ICU were randomized to video vs direct laryngoscopy.

## Study Design and Methods

We conducted a secondary analysis of the DEVICE trial. The DEVICE trial was a randomized controlled study that enrolled adults who were critically ill undergoing intubation at 17 sites in the Pragmatic Critical Care Research Group, including 7 EDs and 10 ICUs.^9^ All adults undergoing tracheal intubation in a participating ED or ICU were eligible except patients who were known to be pregnant or serving a prison sentence and patients for whom the clinician performing the procedure determined that the use of a video laryngoscope or a direct laryngoscope on the first attempt was either required or contraindicated. Patients were enrolled and randomized by treating clinicians or a delegate using sequentially numbered, opaque envelopes. Clinicians were allowed to exclude patients when the urgency of the clinical situation and available team resources precluded safe completion of trial procedures, but clinicians were also allowed to enroll patients undergoing emergency intubations, including patients in cardiac arrest, when safe completion of trial procedures was feasible. In the DEVICE trial, a total of 1,417 patients were assigned randomly in a 1:1 ratio to use of video or direct laryngoscopy. This secondary analysis of a de-identified data set represented nonhuman subjects research (Institutional Review Board Identifier: 160158); secondary review and concurrence of nonhuman subjects research was performed by the Department of Defense Office of Human Research Oversight.

The current secondary analysis of the DEVICE trial included patients (1) for whom the indication for intubation was cardiac arrest, (2) who had experienced a cardiac arrest before initiation of the intubation procedure as reported by the clinician performing the tracheal intubation procedure, and (3) for whom cardiac arrest was listed as an active problem at time of intubation. The exposure of interest was randomized trial group assignment (video laryngoscope group vs direct laryngoscope group). The primary outcome was the same as the original trial: successful intubation on the first attempt, defined as placement of an endotracheal tube in the trachea with a single insertion of a laryngoscope blade into the mouth and either a single insertion of an endotracheal tube into the mouth or a single insertion of a bougie into the mouth, followed by a single insertion of an endotracheal tube into the mouth. All sites used either colorimetric or waveform capnography to confirm successful intubation. Additional outcomes included the duration of laryngoscopy (defined as the time from initial laryngoscope insertion to successful endotracheal tube placement), Cormack-Lehane grade of view, death within 1 hour after intubation, and death by 28 days after intubation.

The primary analysis of the primary outcome was an unadjusted intention-to-treat comparison of successful intubation on the first attempt between patients randomized to the video laryngoscope group and patients randomized to the direct laryngoscope group, using a χ^2^ test. An unadjusted analysis was chosen as the primary analysis because the treatment that patients received (video laryngoscopy vs direct laryngoscopy) was determined by randomization in the parent DEVICE trial and was not subject to confounding, and an unadjusted analysis was the primary analysis of the parent DEVICE trial. To account for relevant covariates, we performed an additional analysis using a generalized linear mixed-effects model using a logit link function with the primary outcome as the dependent variable, study site as a random effect, and fixed effects of study group and the following prespecified baseline covariates: age, sex, BMI, operator experience quantified as the operator’s total number of prior intubations, and location of intubation (ED vs ICU). In adjusted analyses, missing data for covariates was imputed using multiple imputation. All variables that were included in prespecified statistical models also were included in the imputation model. Variables used in the imputation model included: age, sex, BMI, the operator’s prior experience intubating with a video laryngoscope (number of intubations), study site, whether the site was an ED or ICU, and randomized treatment group assignment (direct laryngoscope or video laryngoscope). Continuous variables were modeled assuming a nonlinear relationship to the outcome using restricted cubic splines with between 3 and 5 knots. Duration of laryngoscopy was reported as a mean (SD) and was analyzed using a *t* test. Categorical variables were expressed as numbers (percentages) and were compared using a χ^2^ test.

The DEVICE trial did not capture data regarding whether patients were actively receiving CPR at the time of intubation, and patients included in this secondary analysis could have been undergoing intubation either during CPR or after return of spontaneous circulation. To understand whether the results would have been different if limited to patients receiving CPR at the time of intubation, the primary analysis was repeated among patients who had experienced cardiac arrest and were not administered any sedation, which was used as a surrogate for ongoing CPR.

## Results

Among the 1,417 patients in the DEVICE trial, 113 patients (7.9%) experienced cardiac arrest before intubation. Of these, 87.6% of patients were intubated in the ED and 12.4% of patients were intubated in the ICU. A total of 48 patients were randomized to the video laryngoscope group and 65 patients were randomized to the direct laryngoscope group. Baseline characteristics, including age, sex, race or ethnic group, BMI, and anticipated level of difficulty were similar between groups (Table 1, e-Table 1). Most patients in the video laryngoscope group were intubated with a standard geometry blade (e-Table 2).

**Table 1:** Patient Characteristics

Characteristic	Video Laryngoscopy (n = 48)	Direct Laryngoscopy (n = 65)
Age, y	59 (46-69)	60 (49-68)
Female sex	17 (35.4)	21 (32.3)
Racial or ethnic group^a^		
White, non-Hispanic	29 (60.4)	27 (41.5)
Black, non-Hispanic	9 (18.8)	19 (29.2)
Hispanic	4 (8.3)	10 (15.4)
Other^b^	4 (8.3)	7 (10.8)
Not reported	2 (4.2)	2 (3.1)
BMI^c^	30.5 (25.2-34.7)	26.6 (23.6-32)
Location of intubation		
Emergency department	43 (89.6)	56 (86.2)
ICU	5 (10.4)	9 (13.8)
Operator experience: No. of prior intubations	50 (31-84)	50 (35-100)
Operator training level		
Resident	42 (87.5)	53 (81.5)
Fellow	5 (10.4)	9 (13.8)
Attending	0 (0)	1 (1.5)

Data are presented as No. (%) or median (interquartile range).

a Reported by patients or their surrogates as part of clinical care and collected from the electronic health record by research personnel using fixed categories.

b Includes Asian, American Indian or Alaskan Native, and Native Hawaiian or other Pacific Islander.

c Missing for 9 patients in the video laryngoscopy group and 9 patients in the direct laryngoscopy group.

All 48 patients (100.0%) in the video laryngoscopy group received a video laryngoscopy on the first laryngoscopy attempt; 62 of the 65 patients (95.4%) in the direct laryngoscopy group received a direct laryngoscope on the first laryngoscopy attempt. A complete view of the glottis (grade 1 on the Cormack-Lehane grading scale) was reported in 70.8% of the patients in the video laryngoscopy group, as compared with 46.2% of the patients in the direct laryngoscopy group (absolute risk difference, 24.7 percentage points; 95% CI, 5.2-44.2 percentage points; *P* = .01) (Table 2, e-Table 3).^18^

**Table 2:** Outcomes

Outcome	Video Laryngoscope Group	Direct Laryngoscope Group	Absolute Risk Difference or Mean Difference (95% CI)	*P* Value
Overall				
No. of patients	48	65	NA	NA
Successful intubation on the first attempt	40 (83.3%)	42 (64.6%)	18.7 (1.2-36.2)	.03
Duration of laryngoscopy, s	48.0 (37.3)	98.0 (122.4)	–50.0 (–86.8 to –13.3)	.004
Grade I view	34 (70.8%)	30 (46.2%)	24.7 (5.2-44.2)	.01
Death				
By 1 h	11 (22.9%)	24 (36.9%)	–14.0 (–32.5 to 4.5)	.11
By ICU discharge	33 (68.8%)	45 (69.2%)	–0.5 (–18.2 to 17.3)	.96
By 28 d	35 (72.9%)	46 (70.8%)	2.1 (–16.4 to 20.7)	.80
Among those who did not receive sedation				
No. of patients	24	28	NA	NA
Successful intubation on the first attempt	21 (87.5%)	16 (57.1%)	30.4 (7.8-53.0)	.02
Duration of laryngoscopy, s	44.4 (40.6)	111.1 (115.4)	–66.7 (–15.4 to –118.0)	.006
Death				
By 1 h	8 (33.3%)	20 (71.4%)	–38.1 (–63.3 to –12.9)	.006
By ICU discharge	18 (75.0%)	27 (96.4%)	–21.4 (–40.1 to –2.8)	.02
By 28 d	20 (83.3%)	27 (96.4%)	-13.1 (-29.5 to 3.3)	.11

Data are presented as No. (%) or mean (SD) unless otherwise indicated. NA = not applicable.

**Figure 1:**
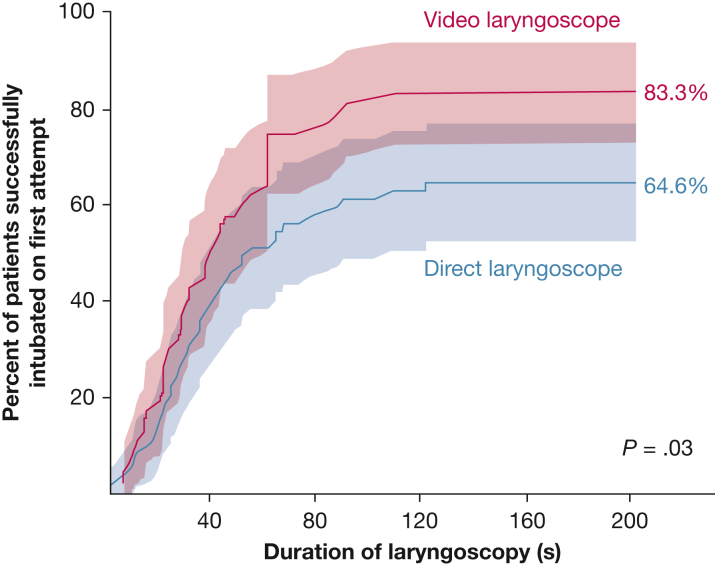
Graph showing the cumulative incidence of successful intubation on the first attempt and 95% CIs (shaded areas) among patients with cardiac arrest in each trial group relative to the time since the initial insertion of a laryngoscope blade into the mouth. Displayed percentages and *P* values are from the primary analysis, rather than the cumulative incidence model. Successful intubation on the first attempt occurred in 40 of 48 patients (83.3%) in the video laryngoscope group and in 42 of 65 patients (64.6%) in the direct laryngoscope group (absolute risk difference, 18.7 percentage points; 95% CI, 1.2-36.2 percentage points; *P* = .03, χ^2^ test).

**Supplementary Figure 1:**
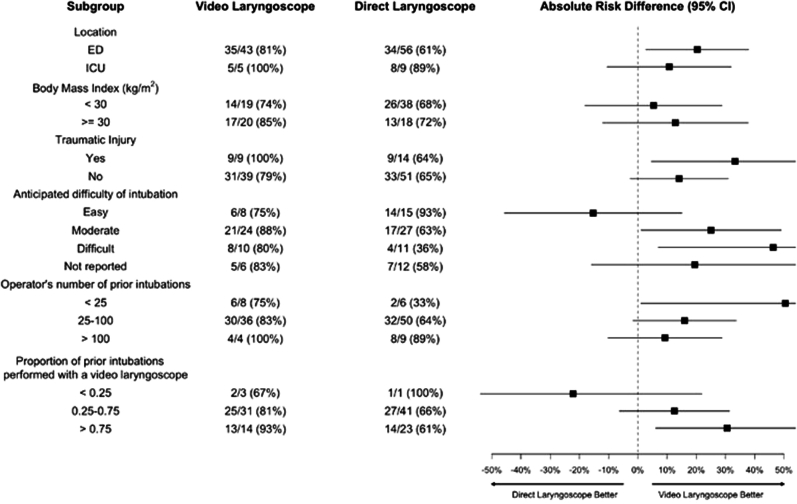


The primary outcome of successful intubation on the first attempt occurred in 40 of 48 patients (83.3%) in the video laryngoscopy group and 42 of 65 patients (64.6%) in the direct laryngoscopy group (absolute risk difference, 18.7 percentage points; 95% CI, 1.2-36.2 percentage points; *P* = .03) (Fig 1). Results were similar in an adjusted analysis with an absolute risk difference of 19.6 percentage points and 95% CI of 13.9 to 25.3 percentage points (*P* < .001) and in an analysis using a Fisher exact test (e-Table 4). The results of the primary outcome in prespecified subgroups are shown in e-Figure 1.

The most common reason for failure on the first attempt in the direct laryngoscope group was inadequate view of the vocal cords (e-Table 5). In the case of failure on the first attempt, a video laryngoscope was the most commonly used device for the final, successful intubation attempt in both groups (e-Table 6). The mean duration of laryngoscopy was 48 seconds (SD, 37.3 seconds) in the video laryngoscopy group and 98 seconds (SD, 122 seconds) in the direct laryngoscopy group (mean difference, –50 seconds; 95% CI, –86.8 to –13.3 seconds; *P* = .004). No cases of intubation failure or cricothyroidotomy occurred in either group. Death within 1 hour of enrollment occurred in 11 of 48 patients (22.9%) in the video laryngoscopy group and in 24 of 65 patients (36.9%) in the direct laryngoscopy group (absolute risk difference, –14 percentage points; 95% CI, –32.5 to 4.5 percentage points; *P* = .11). Death before ICU discharge and death by 28 days after intubation were similar between groups (Table 2).

Of the 113 patients who experienced cardiac arrest before intubation, 48.2% of patients did not receive a sedative before laryngoscopy, a surrogate for patients who had not yet achieved return of spontaneous circulation at the initiation of laryngoscopy. Among these patients, successful intubation on the first attempt occurred in 21 of 24 patients (87.5%) in the video laryngoscopy group and in 16 of 28 patients (57.1%) in the direct laryngoscopy group (absolute risk difference, 30.4 percentage points; 95% CI, 7.8-53.0 percentage points; *P* = .02) (Table 2). The mean duration of laryngoscopy was 44.4 seconds (SD, 40.6 seconds) in the video laryngoscopy group and 111 seconds (SD, 115 seconds) in the direct laryngoscopy group (mean difference, –67 seconds; 95% CI, –15 to –118 seconds; *P* = .006). Death within 1 hour of enrollment occurred in 8 of 24 patients (33.3%) in the video laryngoscopy group and in 20 of 28 patients (71.4%) in the direct laryngoscopy group (absolute risk difference, –38.1 percentage points; 95% CI, –63.3 to 12.9 percentage points; *P* = .006).

## Discussion

Among adults who experienced cardiac arrest before intubation, use of video laryngoscopy increased the incidence of successful intubation on the first attempt by 18.7 percentage points compared with use of direct laryngoscopy. Use of a video laryngoscope also seemed to decrease the duration of laryngoscopy by approximately 1 minute. The finding that video laryngoscopy facilitated intubation on the first attempt and decreased the duration of laryngoscopy are clinically relevant because reducing interruption of chest compressions has been shown to improve outcomes in cardiac arrest. Current guidelines do not recommend the use of a video laryngoscope for tracheal intubation in the setting of cardiac arrest,^12^ and these results may inform future recommendations.

The results of the current study are consistent with the findings of the parent DEVICE trial, which found a 14.3-percentage point increase in successful intubation on the first attempt among a broad population of adults who were critically ill assigned to use of a video laryngoscope. This secondary analysis of the DEVICE trial suggests that the benefit of video laryngoscopy may be even greater for patients who have experienced cardiac arrest (18.7-percentage point increase in successful intubation on the first attempt with video laryngoscopy), particularly those who potentially were receiving CPR (30.4-percentage point increase in successful intubation on the first attempt with video laryngoscopy). The incidences of successful intubation on the first attempt were similar among patients assigned to video laryngoscopy in the cardiac arrest subgroup (83.3%) and among the overall trial cohort (85.1%), whereas successful intubation on the first attempt among patients assigned to direct laryngoscopy was numerically lower in the cardiac arrest subgroup (64.6%) than in the overall trial cohort (70.8%).^9^ This may suggest that the observed benefit of using a video laryngoscope rather than a direct laryngoscope for intubation after cardiac arrest at least in part may be attributable to difficulty intubating with a direct laryngoscope in this population.^3^

Two prior trials have attempted to evaluate the use of a video vs direct laryngoscope during cardiac arrest.^16^^,^^17^ The first trial compared use of a video vs direct laryngoscope during out-of-hospital cardiac arrest among 11 experienced physicians in Japan.^16^ The trial included data on 109 intubations and showed no difference in the primary outcome of time to intubation. Successful intubation on the first attempt was reported to be lower with use of a video laryngoscope (46.4%) compared with use of a direct laryngoscope (75.5%), but the authors noted that although all physicians had at least 3 years of experience, half reported little experience with a video laryngoscope. In the second trial, physicians in 1 ED were randomized to exclusive use of a video laryngoscope or a direct laryngoscope for tracheal intubations occurring during CPR for the duration of the study.^17^ The 14 emergency physicians who participated in the study had performed an average of 93 previous intubations. Among 140 tracheal intubations performed during the study, successful intubation on the first attempt occurred in 94.4% in the video laryngoscope group and 87.0% in the direct laryngoscope group, a difference that was not significant. The duration of chest compression interruption was lower in the video laryngoscope group than the direct laryngoscope group. The differences between the results of this analysis and those of prior trials may be attributable to differences in operator experience. Operators in the DEVICE trial largely were trainees (residents or fellows with a median of 50 previous intubations) with less overall experience than in prior studies. Further, most operators in the DEVICE trial reported similar experience with use of a video and direct laryngoscope, in comparison with prior trials in which operators had performed most of their intubations with a direct laryngoscope. Although operators in the DEVICE trial likely are representative of clinicians who have trained in the era of widespread use of video laryngoscopes, both overall and device-specific experience could affect the relationship between use of a video vs direct laryngoscope and procedural outcomes.

Although current American Heart Association guidelines do not specify whether an endotracheal tube or supraglottic airway should be placed during CPR, prior research on airway management during out-of-hospital cardiac arrest has suggested that initial supraglottic airway placement might improve outcomes compared with tracheal intubation.^19^ Whether these results apply to cardiac arrest being managed in the ED or ICU, where operators have significantly more experience with tracheal intubation, remains unclear. Two ongoing trials soon may provide additional evidence on this question (NCT05520762 and ISRCTN17720457).^20^^,^^21^ Regardless of the initial approach to airway management, most patients who experience in-hospital cardiac arrest in current clinical practice undergo tracheal intubation either during or shortly after CPR, and our results suggest that video laryngoscopy may improve outcomes for these patients.^1^^,^^2^^,^^12^

Strengths of our study include use of a data set from a large, multicenter trial in which randomization of patients to use of a video vs direct laryngoscope prevented confounding by indication. Additionally, a trained, independent observer collected the outcomes of successful intubation on the first attempt and the duration of laryngoscopy.

Our study also has significant limitations. The trial on which this secondary study is based did not include patients who experienced in-hospital cardiac arrest outside of an ED or ICU setting, and the sample size was relatively small, which precludes conclusions regarding whether the effect of video vs direct laryngoscopes differed in key subgroups, such as novice vs experienced operators or planned use of hyperangulated vs standard angulated video laryngoscopes. Operators in the DEVICE trial largely were trainees who reported similar experience with use of a video and direct laryngoscope, so the results may not generalize to highly experienced operators whose prior experience has been limited to a direct laryngoscope. Although all patients had experienced cardiac arrest before intubation, whether patients had achieved return of spontaneous circulation or were still receiving CPR at the time of tracheal intubation was not collected. However, a sensitivity analysis among patients who did not receive sedative medications before laryngoscopy, a potential surrogate for ongoing CPR, showed an even larger difference in favor of the use of a video laryngoscope than the primary analysis, including a significant improvement in survival to 1 hour, a surrogate for return of spontaneous circulation. In addition to gathering data about ongoing CPR and return of spontaneous circulation, future studies would benefit from collecting data on initial rhythm, cause of the arrest, cointerventions, time to return of spontaneous circulation, interruption of chest compressions, and longer-term cognitive and functional outcomes.

## Interpretation

Among adults undergoing tracheal intubation after experiencing cardiac arrest, use of video laryngoscopy increased the incidence of successful intubation on the first attempt and shortened the duration of laryngoscopy compared with the use of direct laryngoscopy.

## Funding/Support

N. J. J. has received support from the 10.13039/100000002National Institutes of Health, the 10.13039/100000030Centers for Disease Control and Prevention, and University of Washington Royalty Research Fund. K. P. S. has received support from the Nation Heart, Lung, and Blood Institute, National Institutes of Health [Grant T32HL087738]. J. A. P. has received support from the National Institute on Aging Grant K23AG073529]. D. W. R. has received support from the Nation Heart, Lung, and Blood Institute, National Institutes of Health [Grant 1K08HL148514]. J. D. C. reports support from the Vanderbilt Trial Innovation Center (U24TR004437-02) and Vanderbilt Center for Learning Healthcare (UL1TR002243-06).

## Financial/Nonfinancial Disclosures

The authors have reported to *CHEST* the following: N. J. J. served on the scientific advisory board for Neuroptics, Inc., as well as on guidelines committees for the International Liaison Committee on Resuscitation and the American Heart Association. S. H. M. received travel support from SharpMed. M. W. S. served as a member of an advisory board for Baxter International, Inc. None declared (A. L. M., K. P. S., E. T. Q., B. I., L. W., M. E. P., B. E. D., S. A. T., D. R.-A., S. G. S., A. A. G., D. W. R., S. G., D. B. P., J. P. G., L. M. S., A. J. L., S. A. G., H. D. W., K. W. G., J. A. P., D. J. V., D. R. J., M. R. W., C. R. B., A. D., A. M., V. K., J. T. H., M. D. A., A. M. J., J. P. W., C. G. H., K. S., A. W. M., B. D. L., S. C. D., W. H. S., T. W. R., J. D. C.).

## Supplementary Data

e-Online Data

e-Online Data
