# Supplementary material for: Video vs Direct Laryngoscopy for Tracheal Intubation After Cardiac Arrest: A Secondary Analysis of the Direct vs Video Laryngoscope Trial
Source: Chest. 2025 Jan 11;167(5):1408–15. doi: 10.1016/j.chest.2024.12.031 (PMC12106960; doi:10.1016/j.chest.2024.12.031)
Supplement: e-Online Data [file mmc1.docx]

**Video versus Direct Laryngoscopy for Tracheal Intubation Following Cardiac Arrest: a Secondary Analysis of the DEVICE Trial**

Supplementary Appendix

**Contents**

**SUPPLEMENTAL FIGURES**

e-Figure 1. Subgroup Analyses of the Primary Outcome 2

**SUPPLEMENTAL TABLES**

e-Table 1. Anticipated difficulty of intubation 3

e-Table 2. Laryngoscope used on first intubation attempt 4

e-Table 3. Glottic visualization 5

e-Table 4. Sensitivity Analysis using a Fishers Exact Test 6

e-Table 5. Reason for failure on the first intubation attempt 7

e-Table 6. Management on the final intubation attempt when successful

intubation on the first attempt did not occur 8

**e-Figure 1. Subgroup Analyses of the Primary Outcome**


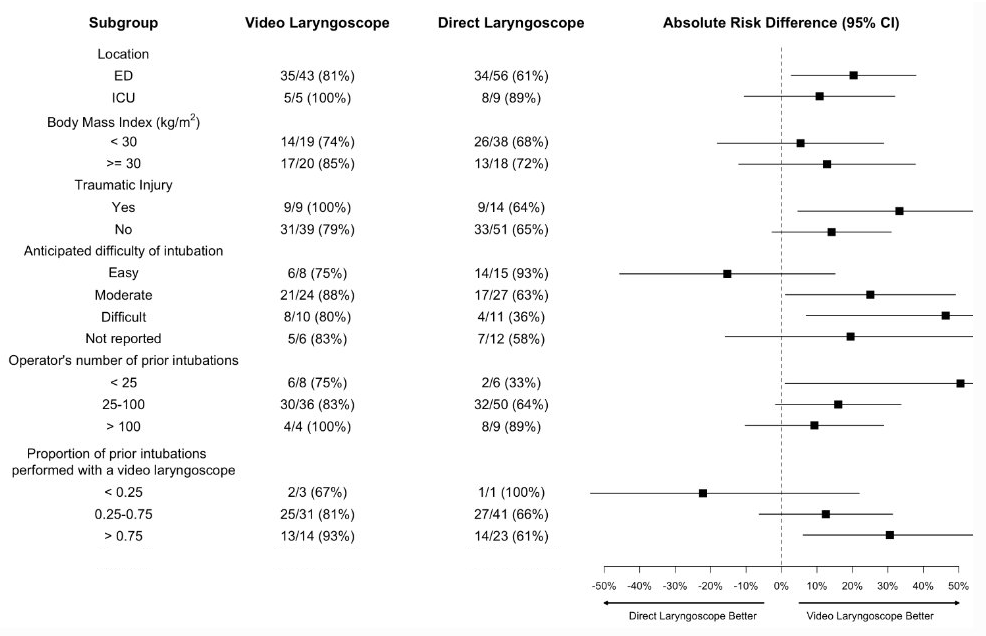

Shown are the absolute risk differences and 95% confidence intervals for the primary outcome (successful intubation on the first attempt) in the video‑laryngoscope group as compared with the direct‑laryngoscope group in each prespecified subgroup. Absolute risk differences were calculated with the use of a generalized linear mixed‑effects model with a random effect for trial site and fixed effects for trial group, the proposed effect modifier, and the interaction between the trial group and the proposed effect modifier. Absolute risk differences of greater than 0 indicate a higher likelihood of successful intubation on the first attempt with use of a video laryngoscope. The body‑mass index is the weight in kilograms divided by the square of the height in meters.

**e-Table 1. Anticipated difficulty of intubation**

| Anticipated difficulty of intubation – no(%) | Video Laryngoscope  (n=48) | Direct Laryngoscope  (n=65) |
| --- | --- | --- |
| Easy | 8 (16.7) | 15 (23.1) |
| Moderate | 24 (50.0) | 27 (41.5) |
| Difficult | 10 (20.8) | 11 (16.9) |
| Not reported | 6 (12.5) | 12 (18.5) |

The anticipated difficulty of intubation was a subjective, global clinical assessment made by the operator before randomization.

**e-Table 2. Laryngoscope used on first intubation attempt**

| Type of laryngoscope – no. (%) | Randomized to Video Laryngoscope (n=48) | Randomized to Direct Laryngoscope (n=65) |
| --- | --- | --- |
| GlideScope LoPro (video, hyperangulated) | 4 (8.3) | 1 (1.5) |
| GlideScope MAC (video, standard geometry) | 10 (20.8) | 0 (0) |
| Macintosh (direct laryngoscope) | 0 (0) | 61 (93.8) |
| McGrath (video, standard geometry) | 1 (2.1) | 0 (0) |
| Miller (direct laryngoscope) | 0 (0) | 1 (1.5) |
| Storz C-MAC (video, standard geometry) | 30 (62.5) | 2 (3.1) |
| Storz D-Blade (video, hyperangulated) | 3 (6.2) | 0 (0) |

**e-Table 3. Glottic visualization**

| Cormack-Lehane Grade of View - no. (%) | Video Laryngoscope  (n=48) | Direct Laryngoscope  (n=65) |
| --- | --- | --- |
| Grade I | 34 (70.8) | 30 (46.2) |
| Grade II | 11 (22.9) | 16 (24.6) |
| Grade III | 3 (6.2) | 13 (20.0) |
| Grade IV | 0 (0.0) | 6 (9.2) |

| **e-Table 4. Sensitivity Analysis using a Fishers Exact Test** | | | |
| --- | --- | --- | --- |
| **Outcome** | **Video Laryngoscope Group**  **(n=48)** | **Direct Laryngoscope Group**  **(n=65)** | **P value** |
| **Overall** |  |  |  |
| Successful intubation on the first attempt – no. (%) | 40 (83.3) | 42 (64.6) | 0.03 |
| Grade I view – no. (%) | 34 (70.8) | 30 (46.2) | 0.01 |
| Death – no. (%) |  |  |  |
| By 1 hour | 11 (22.9) | 24 (36.9) | 0.15 |
| By ICU discharge | 33 (68.8) | 45 (69.2) | 1.0 |
| By 28 days | 35 (72.9) | 46 (70.8) | 0.84 |
| **Among those who did not receive sedation** | **Video Laryngoscope Group (n=24)** | **Direct Laryngoscope Group** **(n=28)** | **P value** |
| Successful intubation on the first attempt – no. (%) | 21 (87.5) | 16 (57.1) | 0.03 |
| Death – no. (%) |  |  |  |
| By 1 hour | 8 (33.3) | 20 (71.4) | 0.01 |
| By ICU discharge | 18 (75.0) | 27 (96.4) | 0.04 |
| By 28 days | 20 (83.3) | 27 (96.4) | 0.17 |

**e-Table 5. Reason for failure on the first intubation attempt**

| Reason – no. (%)* | Video Laryngoscope  (n=48) | Direct Laryngoscope  (n=65) | Absolute Difference (95% CI) |
| --- | --- | --- | --- |
| Inadequate view of the vocal cords | 1 (2.1) | 16 (24.6) | -22.5 (-35.6 to -9.5) |
| Inability to pass the endotracheal tube | 4 (8.3) | 1 (1.5) | 6.8 (-3.4 to 17.0) |
| Inability to pass the bougie | 1 (2.1) | 4 (6.2) | -4.1 (-13.0 to 4.8) |
| Attempt aborted due to patient condition | 0 (0) | 2 (3.1) | -3.1 (-9.1 to 2.9) |
| Other | 1 (2.1) | 2 (3.1) | -1.0 (-7.8 to 5.8) |
| Not reported | 1 (2.1) | 4 (6.2) | -4.1 (-11.2 to 3.0) |

* Reasons for failure were reported by the operator for the 8 patients (16.7%) who failed on the first attempt in the video laryngoscopy group and the 23 patients (35.4%) who failed on the first attempt in the direct laryngoscope group. Patients could have more than one. Confidence intervals were not adjusted for multiple comparisons, so they should not be used for hypothesis testing.

**e-Table 6. Management on the final intubation attempt when successful intubation on the first attempt did not occur**

| Measure – no. (%) | Randomized to Video Laryngoscope  (n=8) | Randomized to Direct Laryngoscope  (n=23) |
| --- | --- | --- |
| **Laryngoscope used on final attempt** |  |  |
| Direct Laryngoscope | 1 (12.5) | 3 (13.0) |
| Video Laryngoscope | 5 (62.5) | 16 (69.6) |
| Unknown | 2 (25.0) | 4 (17.4) |
| Operator different than first attempt | 1 (12.5) | 4 (17.4) |

* Management on the final intubation attempt is reported for the 8 patients (16.7%) who failed on the first attempt in the video laryngoscopy group and the 23 patients (35.4%) who failed on the first attempt in the direct laryngoscope group.
