# Supplementary material for: Video vs Direct Laryngoscopy for Tracheal Intubation After Cardiac Arrest: A Secondary Analysis of the Direct vs Video Laryngoscope Trial
Source: Chest. 2025 Jan 11;167(5):1408–15. doi: 10.1016/j.chest.2024.12.031 (PMC12106960; doi:10.1016/j.chest.2024.12.031)
Supplement: e-Online Data [file mmc2.docx]

**e-Figure 1.** Shown are the absolute risk differences and 95% confidence intervals for the primary outcome (successful intubation on the first attempt) in the video‑laryngoscope group as compared with the direct‑laryngoscope group in each prespecified subgroup. Absolute risk differences were calculated with the use of a generalized linear mixed‑effects model with a random effect for trial site and fixed effects for trial group, the proposed effect modifier, and the interaction between the trial group and the proposed effect modifier. Absolute risk differences of greater than 0 indicate a higher likelihood of successful intubation on the first attempt with use of a video laryngoscope. The body‑mass index is the weight in kilograms divided by the square of the height in meters.
